# Computer-Aided Diagnosis of Pulmonary Nodules in Rheumatoid Arthritis

**DOI:** 10.3390/life12111935

**Published:** 2022-11-20

**Authors:** Anca Emanuela Mușetescu, Florin Liviu Gherghina, Lucian-Mihai Florescu, Liliana Streba, Paulina Lucia Ciurea, Alesandra Florescu, Ioana Andreea Gheonea

**Affiliations:** 1 Department of Rheumatology, University of Medicine and Pharmacy of Craiova, 200349 Craiova, Romania; 2 Department of Medical Rehabilitation, University of Medicine and Pharmacy of Craiova, 200349 Craiova, Romania; 3 Department of Radiology and Medical Imaging, University of Medicine and Pharmacy of Craiova, 200349 Craiova, Romania; 4 Department of Oncology, University of Medicine and Pharmacy of Craiova, 200349 Craiova, Romania

**Keywords:** rheumatoid nodules, computed tomography, computer-aided diagnosis

## Abstract

(1) Background: Rheumatoid arthritis (RA) is considered a systemic inflammatory pathology characterized by symmetric polyarthritis associated with extra-articular manifestations, such as lung disease. The purpose of the present study is to use CAD in the detection of rheumatoid pulmonary nodules. In addition, we aim to identify the characteristics and associations between clinical, laboratory and imaging data in patients with rheumatoid arthritis and lung nodules. (2) Methods: The study included a number of 42 patients diagnosed with rheumatoid arthritis according to the 2010 American College of Rheumatology (ACR)/European League Against Rheumatism (EULAR) criteria, examined from January 2017 to November 2022 in the Departments of Rheumatology and Radiology and Medical Imaging of the University of Medicine and Pharmacy of Craiova. Medical records were reviewed. A retrospective blinded review of CT for biopsy-proven pulmonary nodules in RA using Veolity LungCAD software was performed (MeVis Medical Solutions AG, Bremen, Germany). Imaging was also reviewed by a senior radiologist. (3) Results: The interobserver agreement proved to be moderate (κ = 0.478) for the overall examined cases. CAD interpretation resulted in false positive results in the case of 12 lung nodules, whereas false negative results were reported in the case of 8 lung nodules. The mean time it took for the detection of lung nodules using CAD was 4.2 min per patient, whereas the detection of lung nodules by the radiologist was 8.1 min per patient. This resulted in a faster interpretation of lung CT scans, almost reducing the detection time by half (*p* < 0.001). (4) Conclusions: The CAD software is useful in identifying lung nodules, in shortening the interpretation time of the CT examination and also in aiding the radiologist in better assessing all the pulmonary lung nodules. However, the CAD software cannot replace the human eye yet due to the relative high rate of false positive and false negative results.

## 1. Introduction

Rheumatoid arthritis (RA) is considered a systemic inflammatory pathology characterized by symmetric polyarthritis, predominantly of the small joints, which leads to progressive structural damage of the bone and joint deformity. RA is also associated with extra-articular manifestations, such as lung disease [1]. 

Respiratory disease involves up to 60% of RA patients and can impair the airways, lung parenchyma and pleura. Lung involvement has traditionally been thought to be a late symptom of RA. However, there are growing indications that the lung may be a site of RA onset [2]. 

Rheumatoid nodules are highly specific for RA and occur in 20% of patients, with a predominantly male predisposition and being more common in patients with a smoking history. The presence of subcutaneous nodules is associated with lung nodules in 80% of cases [3].

Several types of nodules may be identified in patients with rheumatoid arthritis such as rheumatoid nodules, lung primary malignancies, metastases, or even benign nodules.

On computed tomography (CT), rheumatoid lung nodules are oval or lobulated, a few millimeters to several centimeters in diameter and frequently peripheral with mid-upper lung disposition. Usually, the nodules are numerous, but they can appear as a single nodule. Calcifications can also be discovered. Nodules may grow in size and number, remaining steady for years or resolving spontaneously [4]. 

On CT scans, malignant nodules are usually larger in size (the risk of malignancy grows proportionally with size), and the margins are ill-defined, irregular in contour or speculated. Malignant nodules may also cavitate, but in a small proportion. A rapid progression in size is usually indicative of either a malignant or an indolent inflammatory disorder [5]. 

On the other hand, metastases tend to have a sharp, smooth margin; are variable in size (the lung lesions appear in a different timeframe and are of different age); and tend to be multiple, located predominantly in the periphery and base of the lungs. In the presence of multiple nodules, lung metastases should initially be taken into account based on the patient’s clinical history and previous imaging findings. 

Benign nodules are usually oval, round or lobulated, with similar sizes and smooth, regular margins [6,7].

Patients with pulmonary rheumatoid nodules are usually asymptomatic. However, symptoms may emerge if nodules cavitate or erode into the pleural space. Nonspecific imaging characteristics can mimic cancer or infection (for example, septic emboli). CT characteristics that usually lead to a diagnosis of rheumatoid nodules over a malignant process include the presence of satellite nodules, a smooth border, calcifications, cavitation and proximity to the pleura [8,9]. 

The ability of a computer system to execute a task without direct involvement or supervision by human intelligence is termed artificial intelligence (AI). There are numerous applications in imaging, including detection tasks, often known as computer-aided diagnosis (CAD) [10].

CAD systems are commonly used to describe the algorithms created for lung nodule diagnosis. They are intended for a variety of applications, including lung segmentation, pulmonary nodule identification and categorization and nodule malignancy prediction.

There have been numerous publications reported on AI algorithms for identifying pulmonary nodules. According to a review article published in 2021 by Schreuder et al., algorithms revealed slightly lower or similar sensitivities to radiologists, at the expense of a considerable rise in the false positive rate [11,12].

The purpose of the present study is to use CAD in the detection of rheumatoid pulmonary nodules. In addition, we aim to identify the characteristics and associations between clinical, laboratory and imaging data in patients with rheumatoid arthritis and lung nodules. Another purpose of our study is to identify the interobserver agreement between CAD and a senior radiologist and to emphasize the benefits CAD systems have to offer. 

## 2. Materials and Methods

### 2.1. Patients

The study included a number of 42 patients diagnosed with rheumatoid arthritis according to the 2010 American College of Rheumatology (ACR)/European League Against Rheumatism (EULAR) criteria, examined from January 2017 to November 2022 in the Departments of Rheumatology and Radiology and Medical Imaging of the University of Medicine and Pharmacy of Craiova. The inclusion criteria were as follows: age >16 years, diagnosis of RA, lung nodules determined by CT examination and proven histologic diagnosis of the lung nodules. The exclusion criteria included the presence of any other rheumatic disease, infection or vasculitis. All patients expressed their agreement to be a part of this study. The study was approved by the local ethics committee of the University of Medicine and Pharmacy of Craiova (Registration no. 208/31.10.2022), according to the European Union Guidelines (Declaration of Helsinki).

### 2.2. Demographic Characteristics and Assessment of Clinical, Laboratory and Imaging Data

Medical records were reviewed. A retrospective blinded review of CT for biopsy-proven pulmonary nodules in RA using Veolity LungCAD software (MeVis Medical Solutions AG, Bremen, Germany) was performed. Imaging was also reviewed by a senior radiologist. 

All patients had undergone physical examination, laboratory tests and CT scan of the lungs, as well as histopathologic examination of an extracted lung nodule, either by thoracoscopy or bronchoscopy.

The evaluated biological parameters consisted of complete blood count (CBC), liver enzymes, serum creatinine, erythrocyte sedimentation rate (ESR) with a normal range < 10 mm/h, C-reactive protein (CRP) with a normal range <5 mg/L, rheumatoid factor (RF) with normal values <14 UI/mL and anti-citrullinated protein antibodies (ACPA) with values >10 U/mL considered positive.

The patients were screened for the presence of subcutaneous rheumatoid nodules. The patient’s pain and severity were assessed with a visual analog scale (VAS) of 100 mm. Standardized scores, such as the diseases activity score 28 with 4 variables (DAS28-4v), were used in order to assess disease activity in the group of patients.

All chest CT scans were performed with intravenous contrast administration on a Siemens Biograph mCT 20 slices device available in the Department of Radiology and Medical Imaging of the University of Medicine and Pharmacy of Craiova. The sequences used for examination were acquired at 1.25 mm slice thickness. The imaging protocol consisted of an initial scanogram, followed by the native acquisition and two postcontrast image acquisitions (arterial and venous phases). The software required image acquisitions in the postcontrast arterial phase, reconstructed in the lung window. After the acquisition of images in an axial plane, sagittal and coronal multiplanar reconstruction (MPR) images were obtained at 1.5 mm slice thickness. 

### 2.3. Statistical Analysis

The statistical analyses of the data were performed using SPSS Software version 20 for Windows. The relationship between the variables was analyzed using the unpaired t-test and the Pearson/Spearman’s coefficient for evaluating correlations. Values less than 0.05 for p were considered to be statistically significant. The summary statistics of the mean ± standard deviation (SD) are presented for continuous variables. The agreement between observers was calculated using cross-tabulation expressed in Cohen’s kappa (κ). The value of kappa was interpreted according to Landis and Koch as follows: <0—poor agreement; 0.0–0.20—slight agreement; 0.21–0.40—fair agreement; 0.41–0.60—moderate agreement; 0.61–0.80—substantial agreement; 0.81–1.00—almost perfect agreement. 

## 3. Results

### 3.1. Baseline Characteristics of the Study Group

The study included 42 patients diagnosed with RA (27 females, 15 males) with a mean age of 62.61 ± 8.36 years. 

Subcutaneous rheumatoid nodules were identified in 23 (54.76%) patients. Rheumatoid factor was positive in 34 (80.95%) of cases, while ACPAs were positive in 19 (45.23%) of patients. Disease activity according to DAS28-CRP was low in 7 (16.67%) cases and moderate and high in 17 (40.47%) and 18 (42.85%) cases, respectively. 

The descriptive parameters of the patients included in the study are presented in Table 1. 

Histopathologic examination of the extracted nodules revealed a diagnosis of rheumatoid nodules in 27 (64.28%) patients. Primary lung malignancy was detected in four (9.52%) patients, out of which one (25%) was categorized as small cell lung carcinoma (SCLC) and three (75%) as non-small cell lung carcinomas (NSCLC). Metastatic nodules were encountered in six (14.28%) patients, while five (11.90%) patients were found to have benign nodules. 

### 3.2. Associations between Clinical, Paraclinical and CT Findings

Mean age ± SD of patients diagnosed with rheumatoid nodules was 63.14 ± 8.52 years, while in the primary malignancy and metastasis groups, it was 64.5 ± 4.79 years and 64.83± 9.06 years, respectively. 

The mean number of rheumatoid pulmonary nodules was of 6.85 nodules per patient. 

Patients with pulmonary rheumatoid nodules were associated with ACPAs positivity (*p* = 0.003), the presence of subcutaneous rheumatoid nodules (*p* = 0.027), younger age (*p* < 0.001) and high disease activity (*p* = 0.034). Higher disease activity was associated with the presence of pulmonary rheumatoid nodules (*p* = 0.048). The presence of rheumatoid lung nodules was also associated with longer disease duration (*p* = 0.043)

Smoker status (*p* < 0.001) and older age (*p* = 0.023) were associated with primary lung malignancy.

### 3.3. Characteristics of the Nodules on the CT Scans

The diameter and margins, as observed by the software and radiologist, are depicted in Table 2. The diameter of the nodules was divided into three categories according to the Fleischner Society’s pulmonary nodule recommendations. 

A nodule detected by the radiologist and not by CAD was considered a false negative result. A nodule detected by CAD which was not actually a nodule was considered a false positive result. The nodules, regardless of the histopathologic type, detected by consensus of CAD and radiologist were considered true nodules. However, the final decision belonged to the radiologist, who was able to discriminate better between nodules and other structures such as fibrosis or broncho-vascular structures. 

Out of the 481 possible nodules detected by either CAD or radiologist, 479 were scored as true nodules by consensus of CAD and radiologist. CAD detected a total of 481 nodules, out of which it missed a total of 8 true nodules (1.67%) and reported another 12 positive nodules (2.49%) which were rejected by the radiologist because they were considered false positive (Table 3). 

**Figure 1:**
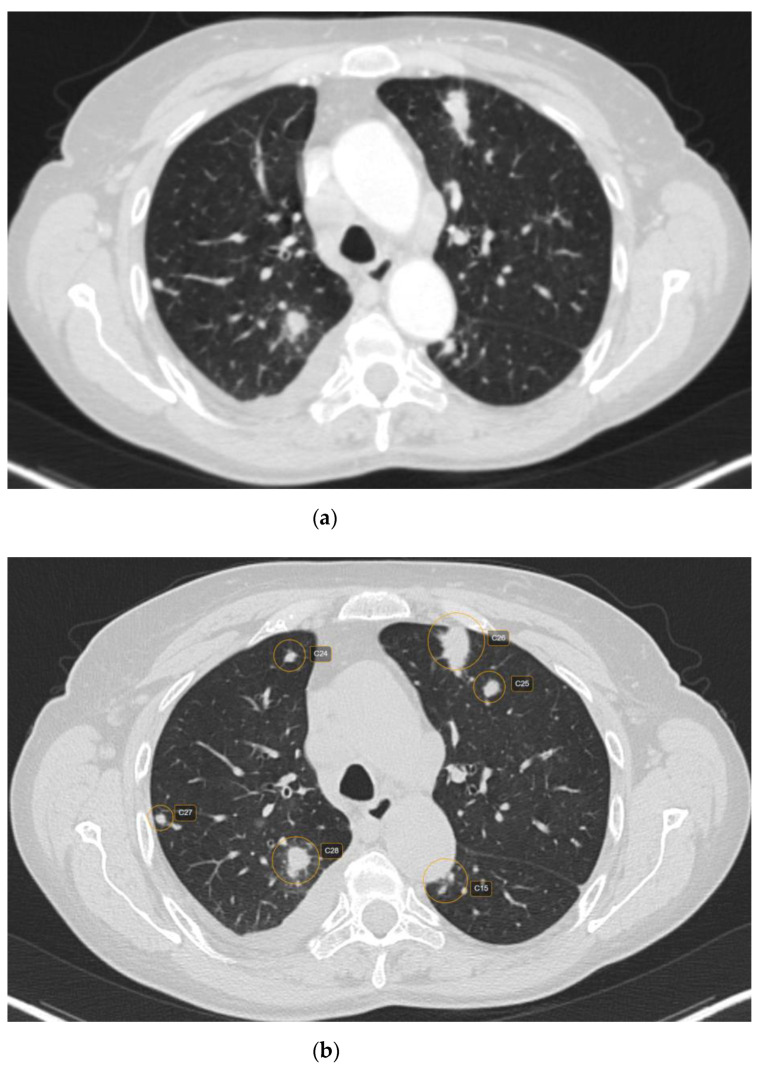
CT scan (lung window) showing raw CT images (**a**) versus marked CT images using CAD (**b**).

**Figure 2:**
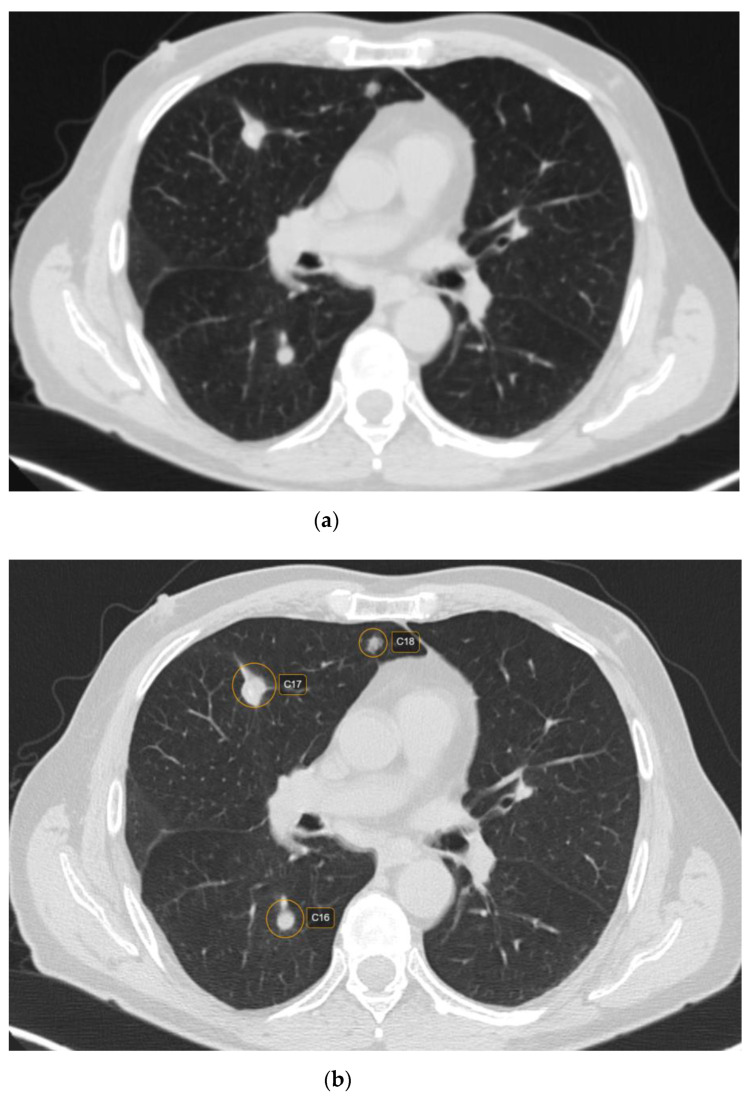
CT scan in lung window depicting lung nodules on raw CT images (**a**) versus marked CT images using CAD (**b**).

Out of 481 possible nodules detected by CAD, 12 were rejected by the radiologist, being considered false positive. The main reasons for false positives included fibrosis—five (41.66%) nodules; broncho-vascular structures—six (50%) nodules; and artifacts due to respiratory motions—one (8.33%) nodule. The majority of the false positive nodules were located in the parahilar area—seven (58.33%) nodules. Regarding the size of the false positive nodules, four (33.33%) had <6 mm, seven (58.33%) had between 6 and 8mm and one (8.33%) had > 8 mm (Figure 1 and Figure 2).

**Figure 3:**
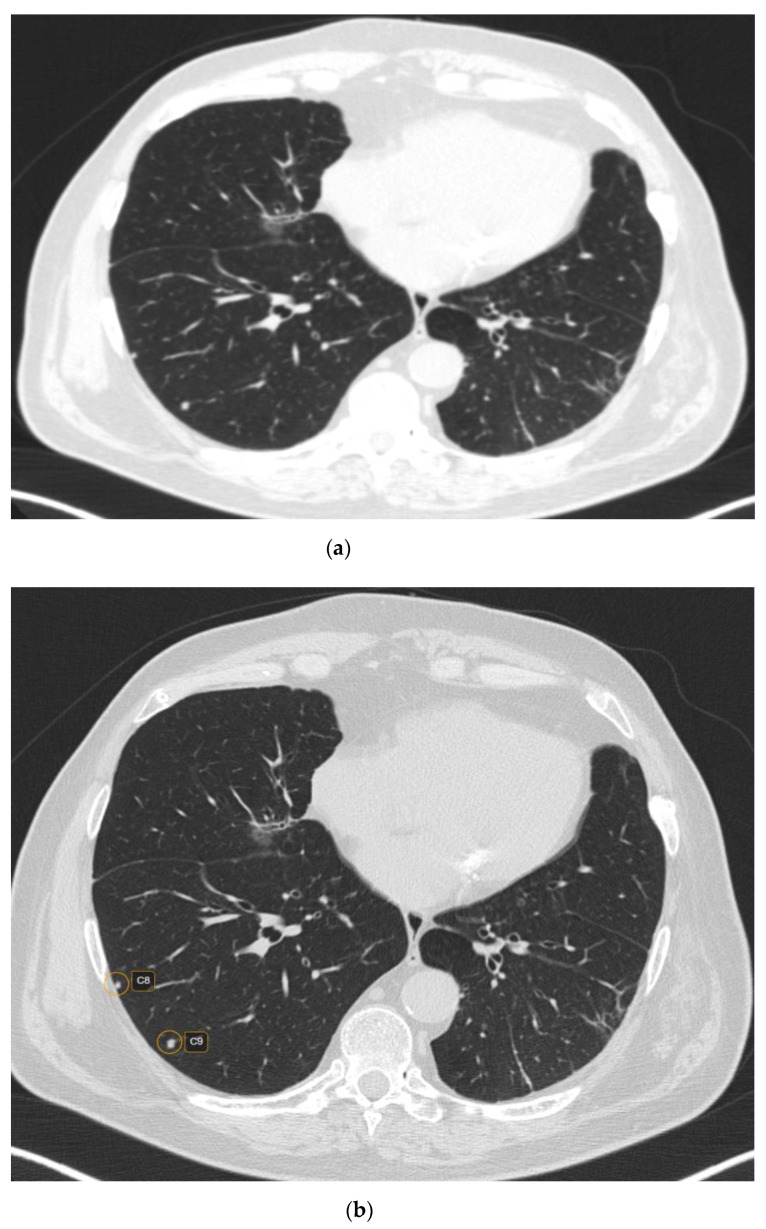
CT scan (lung window) showing raw CT images (**a**) versus marked CT images using CAD (**b**).

CT characteristics of the pulmonary rheumatoid nodules included smooth margins (*p* < 0.001) and a predominantly subpleural location (*p* = 0.045) (Figure 3). In addition, 87 (47.02%) nodules were cavitating, while calcifications were rare, evidenced in 18 (9.72%) nodules. 

Out of the patients with pulmonary rheumatoid nodules, 23 (85.18%) were RF positive and 19 (70.37%) had ACPAs positivity.

### 3.4. Detection Time 

The mean time of detecting the lung nodules using CAD was 4.2 min per patient, whereas the detection of lung nodules by the radiologist was 8.1 min per patient. This resulted in a faster interpretation of lung CT scans, almost reducing the detection time by half (*p* < 0.001).

### 3.5. Interobserver Reliability

The interobserver agreement proved to be moderate (κ = 0.478) for the overall examined cases. The number of nodules detected by CAD and radiologist for each type of nodule (confirmed by histopathologic exam), as well as interobserver agreement are presented in Table 4.

#### Receiver Operating Characteristic Curve 

**Figure 4:**
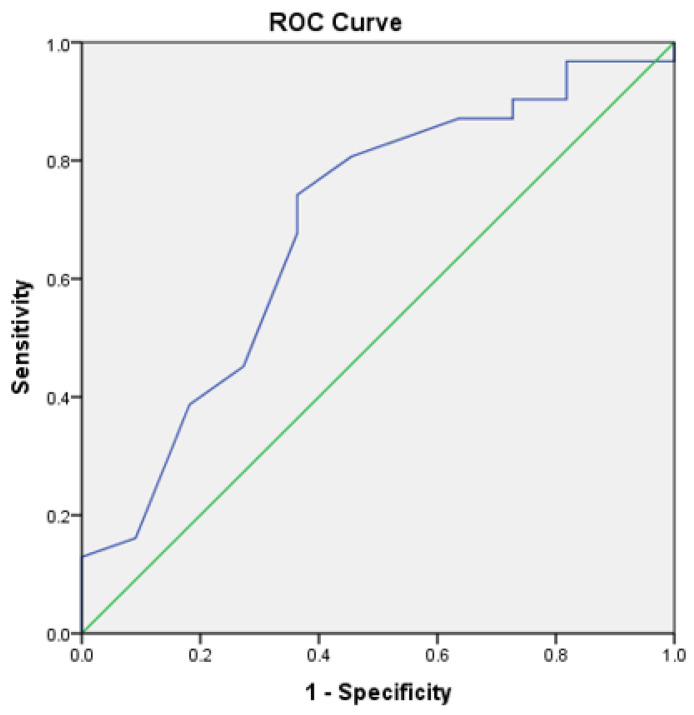
Reader receiver operating characteristic curve for discrimination of pulmonary nodules.

The area under curve (AUC) for CAD determined nodules was 0.68 (95% CI, 0.49–0.87) (Figure 4). The sensitivity and specificity of CAD at 65% threshold was 89.7% and 69.2%, respectively.

## 4. Discussion

In the present study, we described the clinical and imaging characteristics associated with rheumatoid pulmonary nodules in comparison to other types of nodules, either malignant or benign, proven histologically, in patients with rheumatoid arthritis. 

The mean number of rheumatoid pulmonary nodules per patient was 6.85. A study by Koslow et al. in 2018 showed a mean number of 13 rheumatoid lung nodules per patient. This attests to the multiplicity of identified nodules. Predominantly, the nodules were located near the pleura and were typically round with smooth margins, characteristics which have been previously described in literature [13]. 

The presence of rheumatoid lung nodules was associated with seropositivity, younger age and also subcutaneous rheumatoid nodules, in concordance with other studies in literature. A study by Sarikaya et al. in 2022 showed that seropositive RA patients had a higher number of rheumatoid nodules [14]. This proves that RF and ACPAs are risk factors for the development of rheumatoid lung nodules. A study by Natalini et al. in 2021 showed that pulmonary involvement in RA was associated with ACPAs positivity [15]. 

A study by Elsherbiny et al. analyzing the frequency and predictors of extra-articular manifestations in patients with RA showed that the presence of rheumatoid nodules was consistent with higher disease activity and longer disease duration, mostly in older female patients [16]. These findings are in concordance with the results of our study. In addition, literature reviews have emphasized the presence of rheumatoid nodules in patients with positive RF, more severe, with higher disability and high disease activity over a long period of time [17]. 

Innovations in AI breakthroughs are closely linked with CAD system research [18]. However, there are numerous difficulties to applying deep learning algorithms to medical image analysis. For starters, a lack of big training databases is an inescapable impediment in the early stages. According to this principle, needing a bigger number of images to enhance training accuracy, having an extensive training dataset is crucial in deep learning [19]. There several studies have been published in the literature regarding the detection of pulmonary nodules using various CAD systems, with variable sensitivities and specificities, which aided in improving the reader’s diagnostic accuracy [20]. The false positive rate, which reflects the power and reliability of a CAD system, is one of the most essential features of CAD systems. This stage is a key component of the lung nodule detection system, which aids in the detection and early treatment of lung cancer. A. Setio et al. suggested a strategy for reducing false positives using multi-view ConvNets. One way to decrease false positives is to eliminate structures that have less similarity to nodules, which is accomplished by assessing the features of each potential nodule [21]. 

Lee et al. devised a method for identifying lung nodules using a genetic algorithm and pattern matching. False positives were reduced using algorithms based on the features of the nodules discovered. The system was 72% sensitive, with 25.3 false positive detections per case. A total of 98 nodules with dimensions less than 10mm were employed in the system’s validation [22].

Suzuki et al. designed MTANN, an artificial neural network-based pattern-recognition technique, to decrease the frequency of false positives in the diagnosis of lung nodules. This method could process the CT scan directly without the need for segmentation. A sensitivity of 80.3% with 4.8 FP per case was observed when 121 nodules ranging in size from 4 mm to 25 mm were evaluated [23]. 

The Lung Image Database Consortium (LIDC) was founded in 2004 to address one of the most major impediments in the development of CAD systems for the identification of lung nodules: the absence of a database with a large number of examinations. The LIDC was formed by American universities with the objective of establishing and keeping a public database of thoracic CT scans of normal individuals and individuals with different stages of lung malignancy. This information can be used to create, train and evaluate CAD systems for the identification of lung nodules [24].

To enhance sensitivity, Liu et al. advocated dividing pictures into three planes (axial, sagittal and coronal). To segment images and select candidate nodules, the rolling ball algorithm and a dot-enhancement filter were utilized. To eliminate false positives, the nodule properties were retrieved and employed in three support vector machines. They found a sensitivity of 97% and a case rate of 4.3 FP. A drawback of this research is the validation of the system, since it is only evaluated with 32 nodules, 31 of which are single nodules [25].

In the phases of segmentation and identification of lung nodules, Ashwin et al. created a CAD system that used multilevel-thresholding growth and artificial neural networks. This system had a 96% accuracy rate. This method, however, has only been evaluated with 40 patients, including training and validation. Furthermore, the authors did not provide information on the size and location of the nodules analyzed [26].

Kasinathan et al. proposed employing convolutional neural network (CNN) for automated 3D lung tumor identification and categorization. The suggested model was evaluated using the LIDC-IDRI dataset, which contained 850 lung nodule-lesion scans, and the accuracy was 97% [27]. 

Shi et al. suggested a CNN multiscale feature fusion approach for detecting lung nodules. The detection framework is divided into two components: region proposal creation and false positive reduction. VGG16 is the architecture of the CNN model, and trials on the LUNA16 dataset reveal an average sensitivity of 82.62% [28].

The CAD system we used in our research was known to have a rate of two false positive nodule detections per patient and a sensitivity of 85%, thus emphasizing the need for images to be reviewed by a radiologist. Nevertheless, CAD aids the radiologist in detecting the nodules faster, reducing the time needed for the interpretation of a lung CT examination by almost half. In our study, the sensitivity was 90.3%. 

The data in the literature are scarce regarding the CAD of lung nodules in patients with rheumatoid arthritis, thus lacking comparative data regarding the performance of CAD systems in rheumatoid arthritis. 

The limitations of our study included the retrospective design and the low data sample. However, the low number of patients is due to the fact that all the patients included in the study were biopsied, having a certain histopathological diagnosis. This fact aids the identification of certain characteristics of the rheumatoid nodules on the CT scans. Another possible limitation is the fact that our study included patients with pulmonary nodules who were referred for lung biopsy, which is a potential source for selection bias due to the fact that patients without histologic diagnosis of the nodules were not included for comparison. 

## 5. Conclusions

The CAD software is useful in identifying lung nodules, in shortening the interpretation time of the CT examination and in aiding the radiologist in better assessing all the pulmonary lung nodules. However, the CAD software cannot replace the human eye in the near future due to its relative high rate of false positive and false negative results. 

## Figures and Tables

**Table 1:** Demographic and clinical characteristics of patients.

Demographic and Clinical Characteristics (n = 42)	Data
Sex (patients, %)	27 females (64.28%), 15 males (35.72%)
Age (years) (mean, SD)	62.61 (8.36)
Disease duration (years) (mean, SD)	9.2 (3.7)
VAS (mm) (mean, SD)	6.07 (1.53)
ESR (mm/h) (mean, SD)	46.59 (21.98)
CRP (mg/L) (mean, SD)	32.30 (22.35)
RF (patients, %)	34 (80.95%)
ACPAs (patients, %)	19 (45.23%)
DAS28-CRP (mean, SD)	4.74 (1.17)
Smokers (patients, %)	23 (54.76%)

VAS = visual analog scale; ESR = erythrocyte sedimentation rate; CRP = C-reactive protein; RF = rheumatoid factor; ACPAs = anti-citrullinated protein antibodies; DAS = disease activity score; SD = standard deviation.

**Table 2:** Nodule margins and diameter.

	CAD	Radiologist
Nodule diameter		
<6 mm	172	174
6–8 mm	196	198
>8 mm	111	104
Nodule margins		
Spiculated	-	9
Non-spiculated	-	470

CAD = computer-aided diagnosis.

**Table 3:** Nodule detection by CAD and radiologist.

Possible Nodules Detected by CAD or Radiologist	481
Number of nodules considered true nodules detected by radiologist	479
True nodules detected by CAD	473
True nodules detected by radiologist, missed by CAD	8
True rheumatoid nodules detected by CAD	180
True rheumatoid nodules detected by radiologist, missed by CAD	6
Total nodules rejected by radiologist (FP)	12

CAD = computer-aided diagnosis; FP = false positive.

**Table 4:** Number of nodules described by CAD, radiologist and the interobserver reliability for each category of diseases.

CT Findings n = 481/479	CAD n(%)	Radiologist n(%)	CAD vs. Radiologist κ Agreement
Rheumatoid nodules	188 (39.08%)	185 (38.62%)	0.433 Moderate
Primary malignancy	21 (4.36%)	20 (41.75%)	0.636 Substantial
Metastasis	257 (53.34%)	256 (54.35%)	0.455 Moderate
Benign	15 (3.11%)	18 (3.75%)	0.474 Moderate
Overall	481	479	0.478 Moderate

CAD = computer-aided diagnosis.
